# Intelligent pharmaceutical patent search on a near-term gate-based quantum computer

**DOI:** 10.1038/s41598-021-04031-y

**Published:** 2022-01-07

**Authors:** Pei-Hua Wang, Jen-Hao Chen, Yufeng Jane Tseng

**Affiliations:** 1 Graduate Institute of Biomedical Electronics and Bioinformatics, National Taiwan University, No. 1 Sec. 4, Roosevelt Road, Taipei, 106 Taiwan; 2 Department of Computer Science and Information Engineering, National Taiwan University, No. 1 Sec. 4, Roosevelt Road, Taipei, 106 Taiwan; 3 Chunghwa Telecom Co., Ltd, Taipei, 106 Taiwan

**Keywords:** Cheminformatics, Computational chemistry, Quantum information

## Abstract

Pharmaceutical patent analysis is the key to product protection for pharmaceutical companies. In patent claims, a Markush structure is a standard chemical structure drawing with variable substituents. Overlaps between apparently dissimilar Markush structures are nearly unrecognizable when the structures span a broad chemical space. We propose a quantum search-based method which performs an exact comparison between two non-enumerated Markush structures with a constraint satisfaction oracle. The quantum circuit is verified with a quantum simulator and the real effect of noise is estimated using a five-qubit superconductivity-based IBM quantum computer. The possibilities of measuring the correct states can be increased by improving the connectivity of the most computation intensive qubits. Depolarizing error is the most influential error. The quantum method to exactly compares two patents is hard to simulate classically and thus creates a quantum advantage in patent analysis.

## Introduction

Quantum computers operate by quantum coherence and quantum entanglement are expected to achieve unprecedented speedup for some problems compared with classical computers^1^. Some leading candidates like superconducting qubits^2, 3^, trapped ions^4, 5^ and photons^6^ are among the most promising systems for practical quantum computing beyond the reach of modern supercomputers. The most dramatic example is Shor's algorithm for factoring numbers in polynomial time^7^. Another example is Grover's algorithm, which offers a quadratic speedup^8^. Although Grover's algorithm does not provide as spectacular a speedup as Shor's algorithms, it has widespread applicability in search-based methodologies including query-based quantum eigensolver^9^.

**Figure 1:**
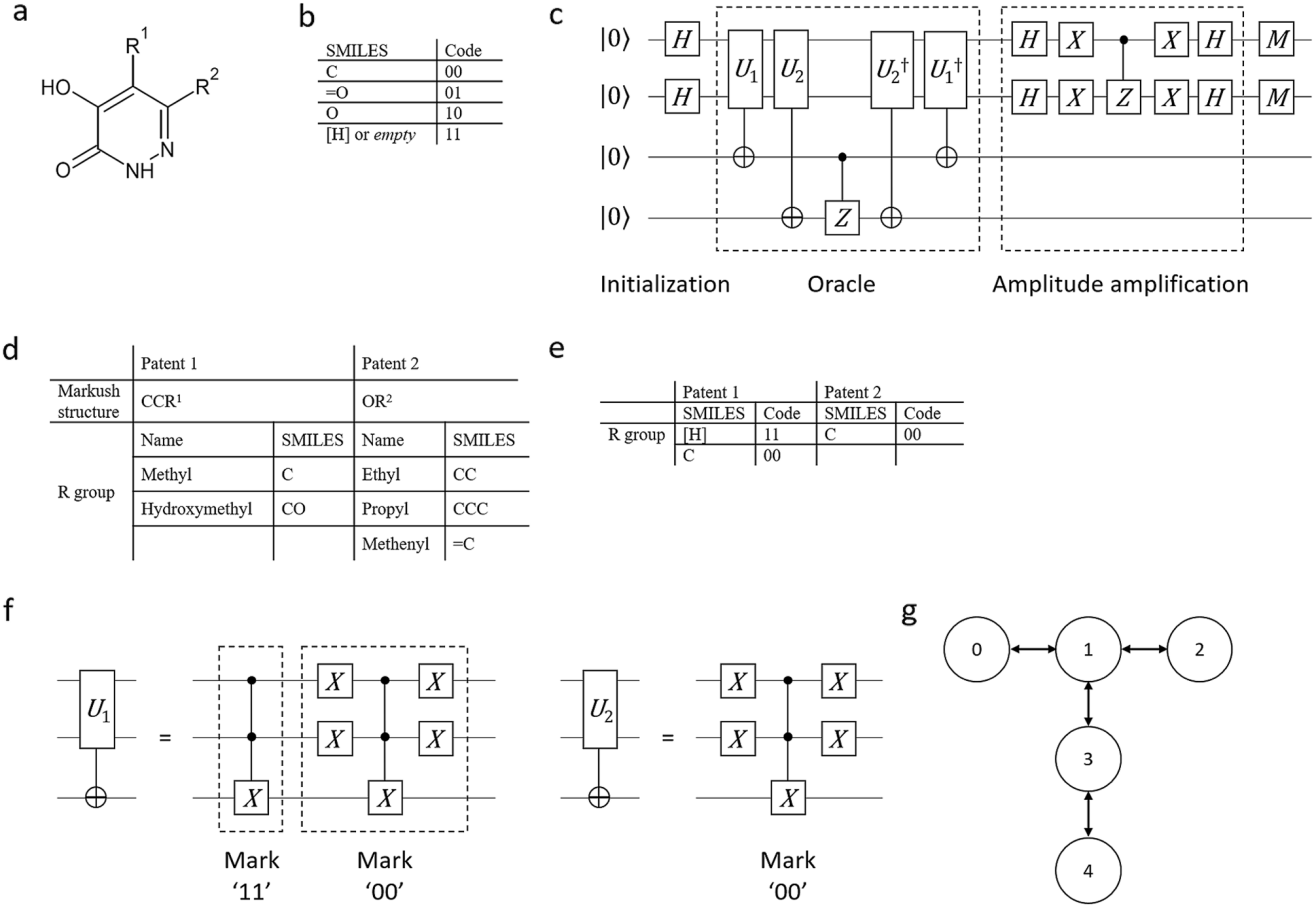
(**a**) The Markush structure of the TAK-831 patent claim. The R group in the structure claim can be defined in a nested manner. In the TAK-831 case, the compounds defined by the Markush structure are infeasible to enumerate. (**b**) The code table to change SMILES symbols into binary quantum states. We used two bits to code a symbol in SMILES notation. An additional code for an empty symbol is introduced to encode the compounds with short SMILES notation. (**c**) The quantum circuit in the 2-data qubit experiment. In the defined case, the Grover iteration only needs to be executed one time. (**d**) The two sets of R groups from two patents to be compared. We use R1 and R2 to denote the variable R groups of the Markush structure in the two patents. R1 has two variable compounds for the R group, and R2 has three variable compounds for the R group. (**e**) The R groups from two patents to be compared using two data qubits. In this simple case, we assume that the core structures of the two patents are the same, and we need to compare only the variate substructures in the R groups. (**f**) The unitary gates used in the 2-data qubit experiment. One of the patent oracles defines two targets 11 and 00 by two MCX gates. The other patent oracle defines only one target by the quantum state 00. (**g**) The layout of ibmq_vigo. The circles indicate qubits along with their ID numbers. The arrows show the connectivity of the quantum computer. Qubit 1, in this case, has the largest connectivity among the qubits.

The current chemical industry relies on patents for product protection. To represent patent claims, the Markush structure is commonly used^10^. The structures are depicted with multiple variable groups, which can all be independent of each other^11^. A Markush structure consists of an invariant core structure and variate substructures called R groups. The combination of R groups gives pharmaceutical patents the power to claim an enormous number of compounds in one statement. Modern patent claims include Markush structures to protect a series of structurally related compounds. A recent example is TAK-831^12^, which can be denoted as in Fig. 1a, which contains a core component and two side-chain R groups, R^1^ and R^2^, leading to the following grammar rule:

R^1^→R.

R^2^→ R*R^3^, R*R^4^, R^4^*R^5^.

R^4^→ R.

R^5^→ R.

R^3^→ R, R*R^6^, R*R^7^.

R^6^→ R.

R^7^→ R.

where R^1^, R^2^, …, R^7^ are all R groups, and R denotes the constant choices of substitution variation. The number of compounds in pharmaceutical patent claims can easily exceed 10^10^.

A modified version of Grover’s algorithm introduced by Long^13^ can search a marked state with 100% certainty by replacing the phase inversion with two-phase rotations and it is shown to be optimal^14, 15^. However, when the number of items in the database is very large, the standard Grover algorithm has already achieved high probability, which is sufficient in most potential applications^13^. In the practical patent search problem, the number of compounds we need to compare can easily exceed 10^10^. Therefore, our algorithm is based on standard Grover algorithm for current practical patent search cases.

Performing structure search is a common routine to check if the developing compounds are already claimed in other patents^16^. Currently, it takes an extremely long time to directly perform one-to-one patent comparisons due to the high computing complexity and the cost of time resulting from the huge number of compounds described in Markush structures. As a result, one has to limit the search with core structure matching and then the side chain instead of comparing two groups of claimed compounds in two patents. It is called substructure search in main chemical patent databases including WIPO^17^ (World Intellectual Property Organization) and SureChemBL^18^. However, when the core structure of one Markush structure is described in the R groups of another Markush structure, the overlapped compounds will be missed by the substructure search algorithm and cause patent infringement in the future. Ideally, the most straightforward patent search is to determine whether query compounds are claimed by a patent. In other words, we want to know in the two sets of input of compounds defined in the Markush structures of patent claims, if there are compounds that exactly match each other.

To determine whether a newly proposed patent has the same compounds as a published patent with a classical computer, a brute-force solution first exhaustively lists all the compounds in the patents and sorts them in canonical order. Afterward, the intersection algorithm can be performed between two sorted compound sets^19^. Simple combinations of scaffolds and R group member lists quickly lead to large numbers of structures^20^. Current chemical patent databases do not actually store all the compounds in the claims since the compound set in a patent is too large, which makes the solution infeasible^21^.

Two common methods developed to effectively retrieve information on Markush structures are molecular fragments and connection tables^22^. For molecular fragment approaches, Fisanick^23^ employs a two-step strategy. First, a large number of structures are screened based on fragments. Then, an iterative atom-by-atom search of the query against the stored structures is performed. For connection table approaches, Holliday and Lynch^24^ transform the stored structures into a reduced graph in which the nodes represent chemically significant groups and the links between these groups are represented by edges. Reduced graphs of the query and stored structures are mapped when they have common attributes. After this filtering step, they are sent to a refined atom-by-atom matching algorithm that checks whether the query is found in the stored structures. These two approaches perform approximate searches and can only be carried out on a specific query structure, so they cannot be applied to the patent comparison problem. We want to know whether any of the sets of structures summarized by a Markush query match any of the structures implied by the Markush structure target in the patent comparison problem.

To perform an exact comparison between chemical compounds with a nonenumerated database, we compare Markush structures by checking a patent rule implemented by an oracle. We demonstrate an implementation of a quantum circuit that can improve the process of searching the intersections of a large number of targets by an algorithm based on Grover’s search^25^.

## Results

### Quantum simulation using 10 qubits on patent comparison to verify the correctness of the circuit

The proposed end-to-end quantum circuit for the drug patent comparison was able to output the correct answer from the quantum simulator, proving that this concept could be used in the future. Here, we considered a simple case for the drug patent comparison to demonstrate the implementation of the proposed quantum algorithm. Two Markush structures from two patents are defined in Fig. 1d. Patent 1 (proposed patent) claims CCR^1^, while Patent 2 (existing patent) claims OR^2^. The goal is to find the compounds claimed by both patents.

Using the code table in Fig. 1b, we transcribed the SMILES notation for each compound as bit strings. Therefore, one quantum state represents one compound. If the number of SMILES symbols in an R group is smaller than the maximum number, "11" will be appended to the end to make every R group the same length. For example, "OCC" will be represented by "10000011" when the maximum length equals 4 SMILES symbols.

Since compound naming is never unique, the same compound can have multiple SMILES strings to describe the same chemical structures. In this example, chemical structures from both patents were encoded in the oracle to include full enumerations of SMILES strings of the chemical structures. In theory, if all the symbols used in SMILES are included in the code table, all the possible substructures of a compound can be encoded.

QRAM can efficiently load a classical database to a quantum computer, mapping N data to log N qubits. In Long and Sun’s study^26^, the scheme utilizing standard 1- and 2-qubit gates can efficiently construct an arbitrary superposed quantum state. The algorithm described by Soklakov and Schack^27^ can also prepare an arbitrary pure state with near 100% certainty based on Grover’s algorithm. Our design has no restrictions on SMILES, meaning that we do not need the SMILES notion to be canonicalized for a chemical structure which is usually a required step. Each SMILES input is processed in a unique computation state with equal probability using a code table that converts every symbol that appears in the input compounds into binary codes and concatenate all symbols to map a compound to a quantum state. Thus, it is not required to encode specific data in quantum superposition states, avoiding the requirements of the QRAM.

The compounds defined by both patents in this example are CCCO and OCCC, which are represented in the quantum states $|00000010\rangle$ = $|2\rangle$ and $|10000000\rangle$ = $|128\rangle$ by the code table, respectively. The simulation without error was performed on the statevector_simulator from the IBM Quantum service. We used 10 qubits, including 8 data qubits and 2 ancilla qubits in this example. The resulting vector states are shown in Table 1. The quantum states $|2\rangle$ and $|128\rangle$ have the highest possibility in the measurement readouts, demonstrating that the quantum circuit correctly computed the expected quantum states. This shows that the search algorithm can determine the compounds in the proposed patent having the same compound as the existing patent, thus potentially infringing on the patent.

**Table 1:** The output vector states in the simulation with 8 data qubits. Quantum states $|2\rangle$ and $|128\rangle$ are the states of the targets.

Quantum state	Final amplitude	Probability of being measured
$|2\rangle$ and $|128\rangle$	0.706	0.498
$|0$-$255\rangle$, other than $|2\rangle$ and $|128\rangle$	0.004	< 0.001
$|\ge 256\rangle$	0	0

The sum of the measured probabilities of these two states is close to 1, showing that the proposed quantum circuit can obtain the correct results.

### Experiments performed on a real physical 5-qubit quantum computer

To test the performance on a real physical device, we took a simpler patent search problem that needed only two data qubits to solve (Fig. 1e). We assumed that the variate substructures of the compounds had been extracted by the converted circuit. The remaining problem was to compare the R groups from the two patents defined in Fig. 1e. If we could find any compound from both patents that had the same R group, then the two patents claimed the same compound. The quantum circuit and detailed description of the unitary gate are shown in Fig. 1c,f, respectively.

The connectivity of the qubits in the quantum computer can influence the performance of the quantum algorithm^28^. The real quantum device “ibmq_vigo” consists of 5 qubits with a T-shaped layout, as shown in Fig. 1g. Qubit 1 connects to three qubits, qubits 0, 2 and 3, while qubit 4 connects only to qubit 3. Due to the high connectivity of qubit 1, we assume that mapping one of the data qubits (q_0_ or q_1_ in Fig. 1c) to physical qubit 1 will result in better performance. The results are shown in Table 2. Among the 1024 shots, 348 shots outputted the correct answer “00” on average when q_0_, q_1_, q_2_, and q_3_ in the quantum circuit were mapped to physical qubits 1, 2, 0, and 3, respectively. In contrast, 303 of the 1024 shots outputted the correct answer on average when q_0_, q_1_, q_2_, and q_3_ were mapped to physical qubits 4, 3, 1, and 0, respectively. This shows that the mapping of virtual qubits to real devices influences the performance.

**Table 2:** The average correct counts and percentages on the real quantum computer ibmq_vigo with two different qubit mapping strategies.

Qubit mapping	Correct count	Correct percentage
T-shaped mapping	348/1024 ± 28	34.0% ± 2.7%
Linear mapping	303/1024 ± 23	29.6% ± 2.2%

The mapping strategy leveraging the advantage of the T-shaped connectivity has better performance than the linear mapping strategy.

### Analysis of the effects on different error types on the quantum processor

The main errors in quantum computers include the thermal relaxation error, depolarizing gate error and readout error. The thermal relaxation error models the decoherence of the quantum computer in losing its quantum coherence in a finite time by processes T_1_ and T_2_. The T_1_ process models a qubit losing its amplitude information, while the T_2_ process models a qubit losing its phase information. The depolarizing gate error models a qubit being depolarized with a certain probability. The depolarized qubit is replaced by a completely mixed state. The readout error models the probability that the measurement result is different from the expected result.

We use a simulator with a noise parameter reported from “ibmq_vigo” to determine the error that has the greatest effect on the correctness of the quantum circuit. The quantum circuit is the same 2-data qubit circuit as the experiment conducted on the physical device (Fig. 1g). The results of the simulation with only one type of error from “ibmq_vigo” are shown in Table 3.

**Table 3:** Comparison of the correctness when one type of error parameter is doubled or halved in the simulation.

Error parameter	Thermal error	Depolarizing error	Measurement error
200%	45.6% ± 1.0%	46.9% ± 1.1%	97.3% ± 0.6%
100%	74.7% ± 0.7%	65.1% ± 1.2%	98.7% ± 0.3%
50%	78.7% ± 1.5%	80.5% ± 0.8%	99.5% ± 0.2%

Reducing the depolarizing error improves the correctness the most.

Depolarization alone lowers the correctness to less than 65.1%. Depolarizing error is the largest source of error in “ibm_vigo”. The second largest is thermal error. Readout error affects the correctness the least.

To investigate how the correctness is influenced when the rates of the different types of error change, we double and halve the error parameters. The results in Table 3 show that reducing the depolarizing error rate improves correctness the most, giving guidance in selecting the quantum computer to increase the correctness of the proposed quantum circuit.

## Discussion

With the proper mapping of chemical structure transformation as the inputs and the proposed algorithm implemented by quantum circuits, a quantum computer can improve the drug patent comparison performance compared to the classical approach. We demonstrated how to map a drug patent comparison problem to a quantum circuit, improving the identification of conflict patent efficiency with a quadratic speedup by using an algorithm based on Grover’s algorithm.

The circuit size is crucial to the quantum circuit design. In the two-data qubit example, the quantum circuit comprises 70 single qubit gates and 20 CNOT gates. The greatest difference in the proposed quantum circuit as the qubit size changes is the MCX gates. The MCX gates are transformed from the defined unitary matrix, and their size can expand quickly. For instance, MCX gates with 3 qubits comprise 10 single-qubit gates and 6 CNOT gates, while MCX gates with 8 qubits comprise 255 single-qubit gates and 254 CNOT gates. With the technique for reducing circuit size mentioned in the methods section, the qubit numbers in the MCX can be reduced while introducing other side effects into the new quantum circuit. Future work will analyze which quantum circuit design can perform better in real quantum computers.

It is obvious in this work that depolarizing error affects the correctness two times more than thermal error, whereas the measurement error slightly lowers the correctness. As our algorithm relies on oracles that flip the phase of target quantum states, it is quite reasonable that depolarizing error that disperses the tagged states to other states is so influential to the measurement outcome. The correct states of small-scale circuits tested on the quantum processor can barely be recognized. However, if the circuit includes more gates or qubits to deal with more complicated cases, all the error will certainly become larger. This is the reason why only short circuits with a small number of qubits are applicable in current quantum computers before efficient error correction codes become available.

To implement the drug patent comparison on a quantum computer, there were three problems we needed to address. First, we needed to express the drug patent using quantum states. We used a code table with empty coding entries to encode all the SMILES symbols in the quantum state. Second, it was difficult to load all the compounds into the quantum state. To address this, we did not limit the input format by applying a Hadamard gate on all qubits. The last problem was to embed the patent comparison into a quantum computer. We used two oracles to flag the detected compounds of the two patents, and we combined them by the intersection operator to flag the intersection of the two patents. The intersection operator could readily incorporate Grover’s algorithm by simply replacing the oracle with the intersection operator.

The quantum–classical hybrid approach is a trend in quantum computing in the noisy intermediate-scale quantum era^29^ (NISQ), which can be used to recover the final state of Grover’s algorithm and hence search for the same targets as Grover’s algorithm^30^. Using a hybrid approach involving both classical and quantum computers to solve the problem is rather practical at this stage. The hybrid approach needs to perform only a short sequence of operations on the quantum computer and can offload some amount of the computation to the classical computer^31, 32^. This makes the computation robust to quantum errors and requires little coherence time. The approach of performing computation only on a quantum computer can lead to a long sequence of operations, making it impossible for the quantum circuit to compute the correct answers due to the accumulated error. Therefore, quantum circuit implementations of quantum computer-only approaches are currently impractical.

However, there is no proof confirming that the quantum–classical hybrid algorithm has better search performance than Grover’s search algorithm with a variational quantum eigensolver. Grover’s search algorithm is optimal regarding oracles, since it has been shown that given an oracle, any algorithm has to apply the oracle at least as many times as Grover’s algorithm does^33^. It is not clear whether optimization methods can achieve better efficiency than oracle methods for unstructured search problems. Therefore, we adopt the original Grover search approach to address the drug patent comparison problem.

The performance of a quantum circuit deployed on a real quantum device is reported. By leveraging the connectivity feature of the quantum computer, the performance of the same quantum circuit can be improved. Furthermore, we analyzed three main sources of error for the quantum computer and found that the depolarizing gate error has the greatest influence on the correctness of the result and that reducing the depolarizing gate error can improve the correctness of the quantum circuit more than reducing the other errors.

With a more powerful and fault-tolerant quantum computer, a more complex patent comparison method can truly be implemented. Although theory and implementation are currently far apart in quantum computing, the development of quantum computers is very fast, similar to the history of semiconductor transistor technology scaling. IBM has released their quantum computer road map, aiming to double the quantum volume of quantum processors annually, and they plan to debut a quantum processor with more than 1000 qubits in 2023^34, 35^. The logical qubits consist of at least five physical qubits to counteract arbitrary single-qubit errors^36^. More qubits in the quantum processor also means that quantum error correction can be implemented to build logical qubits, and arbitrarily long quantum computations can be performed reliably^37^.

We used a code table to encode the SMILES symbols of the compounds. With more qubits available, larger compounds can be compared. There is a milestone at 50 qubits with a circuit depth of approximately 40 on quantum processors since it is beyond what can be simulated by the most powerful classical computer^38, 39^. That is, the proposed circuit can be applied in practical chemistry usage in the near future. The exact comparison for patent analysis builds upon the realization of a search-based method with a constraint satisfaction oracle that is hard to simulate classically is an important part of creating a quantum advantage. Hybrid techniques such as our algorithm should be able to adapt to similar questions quickly and easily, and be readily used.

## Methods

We propose a quantum circuit for drug patent comparison, as shown in Supplementary Fig. S1. The input of the circuit is the SMILES notation of all the compounds encoded by the code table. The quantum circuit then carries out a set intersection between the two patent formulas by an algorithm based on Grover’s search. We use two oracles to identify the two patents and combine them to build the quantum intersection oracle; therefore, it can be applied to identify the conflicts of the newly proposed patent with the existing patent.

The oracle specified here can detect drug patents such as TAK-831 and other patent claims. We take some simple examples for formula overlap detection, but it can easily be implemented for more complex formulas defined in drug patents in a similar way.

The programming language we used to implement the quantum circuit is Python with the qiskit library^40^. The MCX gate in this study is implemented using an operator that directly transforms a unitary matrix into gates. Every MCX gate with different numbers of qubits is generated separately by a matrix with the corresponding size.

### Quantum circuit design of the oracle recognizing patent members

The oracle in the quantum circuit recognizes members of the patent from the input SMILES by checking each of the rules defined in the patent. It can be designed and implemented by a classical circuit, and we can convert it to a reversible circuit on a quantum computer since any arbitrary classical circuit can be simulated by an equivalent reversible circuit. Descriptions of this process are given in^41, 42^, and we refer the reader to those works for a more detailed introduction.

There are some tricks that can be applied here to perform conversion work in practice. First, to maintain the reversibility requirement of the quantum circuit, we can modify the output of the classical circuit to include a copy of the input. Another trick is to remove the interference of the ancilla qubits. In classical computing, it is common to use a temporary space to record intermediate results. At the end of the computation, the classical computer can simply discard these results without any side effects, while in the quantum computer case, these intermediate results will interfere with the working qubit. Therefore, it is necessary to remove the interference of the ancilla qubits at the end of the computation. Consider the case in which the classical algorithm performs the following process:

$${V}_{f}\left|x,\mathrm{0,0}\right.\rangle =|x,f\left(x\right),g\left(x\right)\rangle$$

The third register is used as a temporary space, which is common in classical algorithms.

To remove the interference of the ancilla qubits, we need another blank variable to copy the information of f(x), which will become the final output of f(x). This transforms the state in the following way:

$$\left|x,f\left(x\right),g\left(x\right), 0\right.\rangle \to |x,f\left(x\right),g\left(x\right),f\left(x\right)\rangle$$

To remove the interference of the ancilla qubit, we can simply apply ${{V}_{f}}^{\dag}$ to it to undo the original computation:

$$\left|x,f\left(x\right),g\left(x\right), f\left(x\right)\right.\rangle \to |x,0, 0,f\left(x\right)\rangle$$

This yields the desired transform ${U}_{f}$.

By a similar approach, we can build a quantum oracle with classical circuit design.

### Quantum circuit design of formula overlap detection

Let the input to the formula overlap detection module have *n* qubits. Then, the input to the quantum intersection oracle inside the module is denoted as $|n\rangle$, and the output of the intersection oracle is ${(-1)}^{{f}_{M\cap K}(x)}|n\rangle$, where ${f}_{M}$ and ${f}_{k}$ are oracles of the function that determine whether the compound is in patent M or patent K. The oracles used in Grover’s search algorithm for intersection consist of three parts, as shown in Supplementary Fig. S2. The first part specifies the compounds in the patents by using an MCX gate on the corresponding target qubit. The second part is set intersection. We use the controlled-Z gate on both target qubits to flip the phases of the states in the intersection. The third part is to reverse the circuit from the first part to disentangle the data qubits and target qubits.

The quantum circuit for formula overlap detection is built on the basis of Grover’s algorithm, as shown in Supplementary Fig. S3.

The formula overlap detection method is configured as follows:

Input: *n*-bit string representing the SMILES of a compound.

Output: the overlapping compounds, if present.

Qubits: *n* qubits and 2 ancilla qubits. *n* is the number of qubits capable of describing the data and is determined by the length and the number of usable symbols in the input text string. For example, a 3-character text string with 4 different symbols can be represented by $n=3*\lceil{\mathrm{log}}_{2}4\rceil=6$ qubits. Ancilla qubits are used to enable the intersection oracle to flip the phases of the overlapping compounds for the newly proposed patent.

The steps of the formula overlap detection module are as follows:

1. Initialization
  Apply the H gate to all *n* data qubits.
2. Oracles
  (i) Compounds
    Use MCX to assign to the targets.
  (ii) Intersection
    Apply the controlled-Z gate to the target qubits of the two patent oracles. One target qubit is used as the control, and the other target qubit is used as a controlled-Z gate.
  (iii) Untangle the target qubits
    Reverse the circuit in steps (i) to untangle the quantum qubits and data qubits.
    For example, to assign 010 to the patent, apply X gates to the first and third data qubits and MCX to the target qubit using the data qubits as a control. The interference has to be removed by applying X gates to the first and third data qubits again to restore the states of the data qubits. The quantum circuit described here is shown in Supplementary Fig. S4.
3. Amplitude amplification:
  Apply the quantum circuit of the amplitude amplification in the standard Grover search algorithm.
4. Loop Grover’s iteration
  Repeat Steps 2 and 3 *k* times, where *k* is determined by the number of search items in the problem and the number of solutions.
5. Measurement: Measure the outcomes of the data qubits.

In Supplementary Fig. S3, the first and second lines represent the *n* data qubits encoding the input compounds. We use the phase oracle to perform the Grover search algorithm; therefore, a qubit is shown separately to denote the phase flip action that will kick back to the whole entangled state. The unitary operators U_1_ and U_2_ are the oracles of the two patents. The two ancilla qubits are used for the quantum intersection of the two patent oracles. We replace the Toffoli gate with a controlled-Z gate to reduce the qubits needed by one. Note that the complex conjugate of the unitary operator has to be applied in reverse order to remove the interference of the ancilla qubits.

The basic components of the unitary gates of the oracle shown in Supplementary Fig. S4 specify the compounds in each patent. This kind of oracle design requires one MCX to mark a single search item, which can lead to a large circuit size, as shown in Supplementary Fig. S5.

The quantum gate size of the oracle can be designed specifically for the patent to reduce the circuit size. We found some general techniques that can reduce the circuit size, although they have some side effects. For example, we can reduce the circuit by encoding the patent as a 3-SAT problem and then designing a quantum circuit from the 3-SAT problem^43^. For example, if we have three search items $(\overline{A }\overline{B }\overline{C }\overline{D }+\overline{A }\overline{B }\overline{C }D+\overline{A }B\overline{C }\overline{D })$, we can use multiple MCX gates to mark the search items, as shown in Supplementary Fig. S5. We can convert them to a 3-SAT problem as follows:

$$\overline{A }\overline{B }\overline{C }\overline{D }+\overline{A }\overline{B }\overline{C }D+\overline{A }B\overline{C }\overline{D }=\overline{A }\overline{C }\cdot \left(\overline{B }\overline{D }+\overline{B }D+B\overline{D }\right)=\overline{A }\overline{C }\cdot \left(\overline{B }\cdot \left(\overline{D }+D\right)+B\overline{D }\right)=\overline{A }\overline{C }\cdot \left(\overline{B }+B\overline{D }\right)=\overline{A }\overline{C }\cdot ((\overline{B }+B)\cdot (\overline{B }+\overline{D })=\overline{A }\cdot \overline{C }\cdot \left(\overline{B }+\overline{D }\right)$$

Supplementary Fig. S6 shows an example of implementing a 3-SAT binary expression for

$$\overline{A }\cdot \overline{C }\cdot \left(\overline{B }+\overline{D }\right)$$

Gate 1 implements the Boolean functions $\overline{A }$ and $\overline{C }$. Gates 2 and 3 implement the Boolean function $\left(\overline{B }+\overline{D }\right)$ by $\sim (BD)$, and gate 4 performs the AND function for all three clauses. Note that in this case, the technique introduces one more qubit to compute the result. Another technique reduces multiple input MCX gates to fewer inputs^44^. However, this technique may produce more MCX gates than the original technique.

All the quantum experiments and simulations are repeated 10 times to avoid sampling bias. The numbers reported in the results are the averaged counts and percentages with 95% confidence intervals.

## Supplementary Information

Supplementary Information.

## Data Availability

Data sharing is not applicable to this article since no data were generated or analyzed during the current study.

## Code availability

The codes used to perform quantum simulation for noiseless demo and noise analysis can be found in the GitHub repository: https://github.com/wangpeihua1231/quantum_pharmaceutical_patent_search.
