# Author Correction: Intelligent pharmaceutical patent search on a near-term gate-based quantum computer

**DOI:** 10.1038/s41598-022-06175-x

**Published:** 2022-02-01

**Authors:** Pei‑Hua Wang, Jen‑Hao Chen, Yufeng Jane Tseng

**Affiliations:** 1 Graduate Institute of Biomedical Electronics and Bioinformatics, National Taiwan University, No. 1 Sec. 4, Roosevelt Road, Taipei, 106 Taiwan; 2 Department of Computer Science and Information Engineering, National Taiwan University, No. 1 Sec. 4, Roosevelt Road, Taipei, 106 Taiwan; 3 Chunghwa Telecom Co., Ltd, Taipei, 106 Taiwan

Correction to: *Scientific Reports* 10.1038/s41598-021-04031-y, published online 07 January 2022

The Acknowledgements section in the original version of this Article was incomplete.

“Resources at the Laboratory of Computational Molecular Design and Metabolomics and the Department of Computer Science and Information Engineering at National Taiwan University were used in performing these studies. This work was supported by the Neurobiology & Cognitive Science Center at NTU [NTU-CC-109 L892703, NTU-CC-110L890803], TFDA grant number [MOHW109-FDA-D-114-000611, MOHW110-FDA-D-114-000611], and the Ministry of Science and Technology (Taiwan) [MOST 107-2627-E-002-002, 109-2320-B-002-040-, 109-2627-M-002-003-, 109-2823-8-002-010-, 110-2320-B-002-038-].”

now reads:

“Resources at the Laboratory of Computational Molecular Design and Metabolomics, the Department of Computer Science and Information Engineering at National Taiwan University, and the IBM Q Hub at NTU were used in performing these studies. This work was supported by the Neurobiology & Cognitive Science Center at NTU [NTU-CC-109 L892703, NTU-CC-110L890803], TFDA grant number [MOHW109-FDA-D-114-000611, MOHW110-FDA-D-114-000611], and the Ministry of Science and Technology (Taiwan) [MOST 107-2627-E-002-002, 109-2320-B-002-040-, 109-2627-M-002-003-, 109-2823-8-002-010-, 110-2320-B-002-038-].”

The original Article has been corrected.

