# Supplementary material for: Intelligent pharmaceutical patent search on a near-term gate-based quantum computer
Source: Sci Rep. 2022 Jan 7;12:175. doi: 10.1038/s41598-021-04031-y (PMC8742058; doi:10.1038/s41598-021-04031-y)
Supplement: Supplementary file 1 — Supplementary Information. [file 41598_2021_4031_MOESM1_ESM.docx]

**Intelligent pharmaceutical patent search on a near-term gate-based quantum computer**

Pei-Hua Wang^1^, Jen-Hao Chen^2, 3^, Yufeng Jane Tseng^1,2,*^

^1^Graduate Institute of Biomedical Electronics and Bioinformatics, National Taiwan University, No. 1 Sec. 4, Roosevelt Road, Taipei, Taiwan 106

^2^Department of Computer Science and Information Engineering, National Taiwan University, No. 1 Sec. 4, Roosevelt Road, Taipei, Taiwan 106

^3^Chunghwa Telecom Co., Ltd., Taipei, Taiwan, 106

**Supplementary Information**
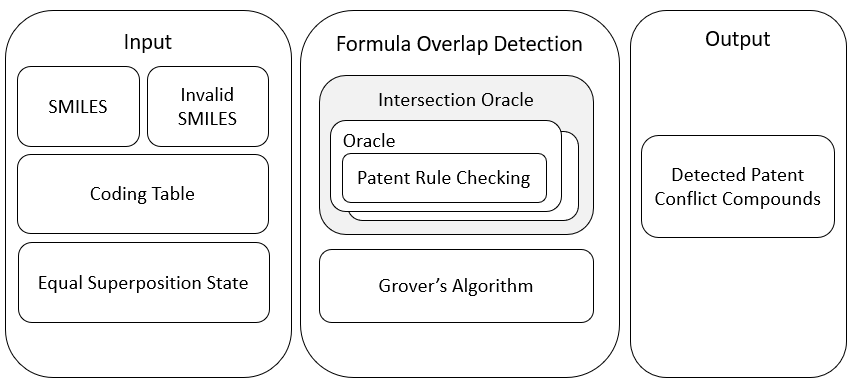


**Supplementary Figure S1.** Overview of the drug patent comparison design. The input is the equal superposition state, which can be interpreted by a code table. The module of formula overlap detection uses Grover’s algorithm to find the overlap of two patents claimed in Markush structures.


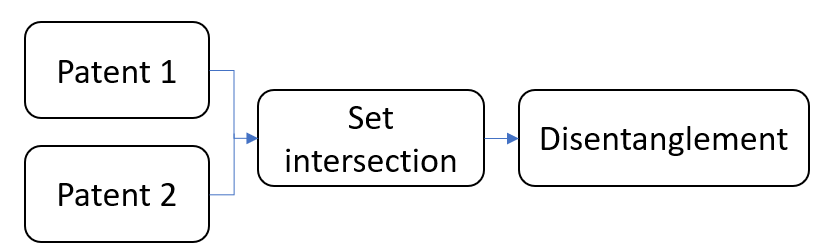


**Supplementary Figure S2.** The oracles used in the formula overlap detection module. The oracles identifying the patent are built by checking all the rules of the patent. The set intersection oracle combines two patent-identifying oracles. The disentanglement process redoes the process to remove the entanglement of the ancilla qubit.


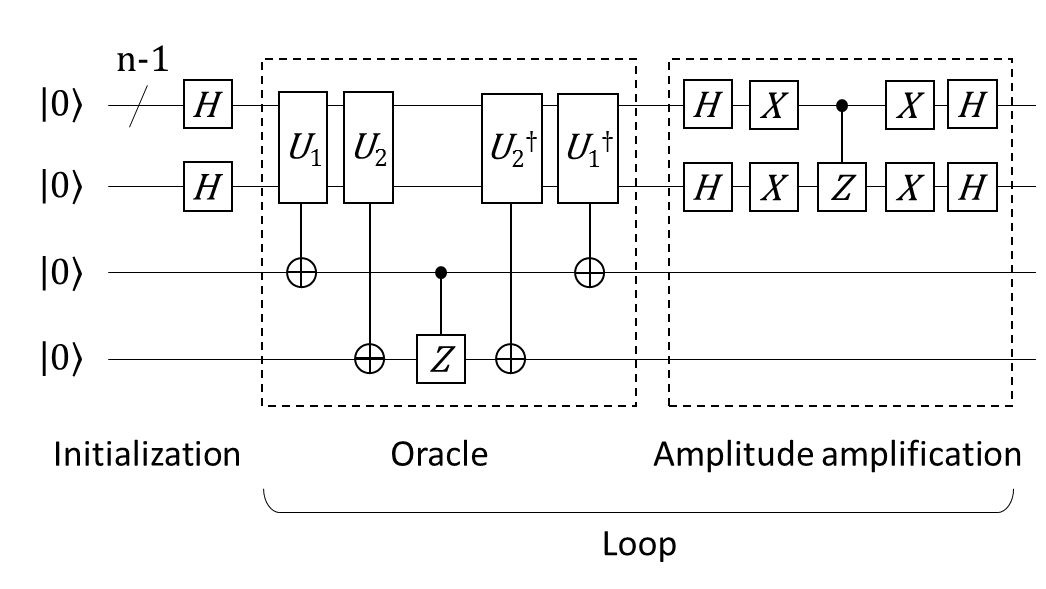


**Supplementary Figure S3.** The quantum circuit in the n data qubit simulation. The oracle and amplitude amplification steps are repeated several times depending on the target count and the size of the search space.


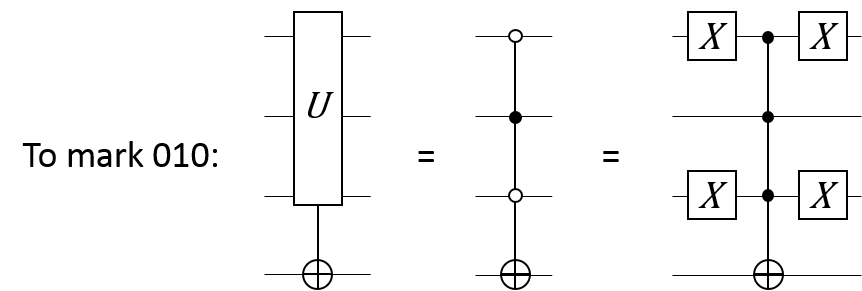


**Supplementary Figure S4.** The unitary gate used to mark the quantum state 010. This is a straightforward method to mark the answer directly with the X gate and MCX gate. Note that after the marking process by the MCX gate, the X gate has to be applied again to remove the entanglement of the X gate.


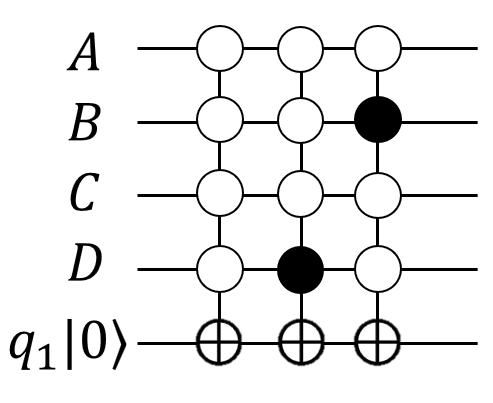


**Supplementary Figure S5.** Multiple MCX gates mark the target items. This example marks the target state of $(\bar{A}\bar{B}\bar{C}\bar{D}+\bar{A}\bar{B}\bar{C}D+\bar{A}B\bar{C}\bar{D})$.


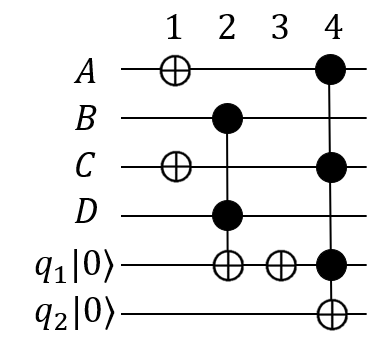


**Supplementary Figure S6.** An example of conversion from a 3-SAT problem to a quantum circuit. The number of qubits in the MCX gate is reduced, while the number of ancilla qubits is increased.
